# Time interval between the completion of radiotherapy and robotic-assisted surgery among patients with stage I–III rectal cancer undergoing preoperative chemoradiotherapy

**DOI:** 10.1371/journal.pone.0240742

**Published:** 2020-10-16

**Authors:** Ching-Wen Huang, Wei-Chih Su, Tzu-Chieh Yin, Po-Jung Chen, Tsung-Kun Chang, Yen-Cheng Chen, Ching-Chun Li, Yi-Chien Hsieh, Hsiang-Lin Tsai, Jaw-Yuan Wang

**Affiliations:** 1 Division of Colorectal Surgery, Department of Surgery, Kaohsiung Medical University Hospital, Kaohsiung Medical University, Kaohsiung, Taiwan; 2 Department of Surgery, Faculty of Medicine, College of Medicine, Kaohsiung Medical University Hospital, Kaohsiung Medical University, Kaohsiung, Taiwan; 3 Division of General and Digestive Surgery, Department of Surgery, Kaohsiung Medical University Hospital, Kaohsiung Medical University, Kaohsiung, Taiwan; 4 Department of Surgery, Kaohsiung Municipal Tatung Hospital, Kaohsiung Medical University, Kaohsiung, Taiwan; 5 Division of Colorectal Surgery, Department of Surgery, Kaohsiung Municipal Hsiaokang Hospital, Kaohsiung, Taiwan; 6 Graduate Institute of Clinical Medicine, College of Medicine, Kaohsiung Medical University, Kaohsiung, Taiwan; 7 Graduate Institute of Medicine, College of Medicine, Kaohsiung Medical University, Kaohsiung, Taiwan; 8 Center for Cancer Research, Kaohsiung Medical University, Kaohsiung, Taiwan; 9 Clinical Pharmacogenomics and Pharmacoproteomics, School of Pharmacy, Taipei Medical University, Taipei, Taiwan; Chang Gung Memorial Hospital at Linkou, TAIWAN

## Abstract

**Background:**

This aim of this study was to evaluate the effects of time interval between the completion of radiotherapy and robotic-assisted surgery on the outcomes among patients with rectal cancer undergoing preoperative concurrent chemoradiotherapy (CCRT).

**Methods:**

In total, 116 patients with stage I–III rectal cancer who underwent preoperative CCRT and robotic-assisted surgery between September 2013 and February 2019 were enrolled. Patients were categorized into two groups based on the time interval: group A (10–12 weeks) and group B (≥ 12 weeks).

**Results:**

Among the 116 enrolled patients, 98 (84.5%) had middle and lower rectal cancers. Two (1.7%) patients underwent abdominoperineal resection with a sphincter preservation rate of 98.3%. Thirty-seven (31.9%) patients had a pathologic complete response (pCR). The circumferential resection margin and distal resection margin were positive in 2 (1.7%) and 1 (0.9%) patients, respectively. Therefore, the R0 resection rate was 97.4%. A total of 24 (22.4%) patients experienced postoperative relapse and 12 (10.3%) patients died; these were slightly more common in group B than in group A (28.8% vs 15.8% and 15.3% vs 5.3%, respectively; both *P* > 0.05); however, this difference was nonsignificant. Three-year disease-free survival (DFS) and overall survival (OS) were 75% and 89%, respectively, among all patients. Non-significant trend of favorable 3-year DFS, 3-year OS, 3-year locoregional control rate and 3-year distant metastasis control rate were observed in group A compared with group B (all *P* > 0.05).

**Conclusion:**

Robotic-assisted surgery after a longer interval is safe and feasible for patients with rectal cancer undergoing preoperative CCRT. The present study’s results suggested that the time interval of 10–12 weeks can be considered because comparable clinical and perioperative outcomes and preferable oncological outcomes were observed for interval of this length. However, future prospective randomized clinical trials are required to verify the present finding.

## Introduction

In the past three decades, the treatment outcomes of rectal cancers have been substantially improved through novel therapeutic modalities and improved surgical approaches. The standard surgical approach for patients with rectal cancer has been total mesorectal excision (TME) surgery, as reported by Heald and Ryall [1] in 1982, because it remarkably improves the clinical outcomes of these patients. MacFarlane *et al*. reported a 5–year locoregional recurrence (LR) rate of 5% among patients receiving monotherapy with TME surgery [2]. However, Tepper JE *et al*. reported a high 5–year LR rate of 14% and poor 5-year overall survival (OS) of 64% among patients with locally advanced rectal cancer (LARC) undergoing curative surgery and postoperative concurrent chemoradiotherapy (CCRT) [3]. A German study reported a marked reduction in LR among patients receiving preoperative CCRT [4, 5], and similar results have been previously reported [6–8]. Therefore, preoperative CCRT has since been the recommended as a standard treatment for patients with LARC.

Because of its downsizing and downstaging effects, preoperative CCRT reportedly serves as a potential treatment modality for patients with LARC to sequentially enhance the potential for R0 resection and the anal sphincter preservation rate [9, 10]. Furthermore, a pathologic complete response (pCR), an indicator of good clinical oncological outcomes, could be achieved through preoperative CCRT in approximately 8%–38% of cases [11–18]. The pCR rate would be affected by the duration of radiotherapy (i.e., short or long) [19–21], the interval between the completion of radiotherapy and surgery [16, 17, 22–28], and the chemotherapy regimen [12–18, 24, 29] in preoperative CCRT. Increased tumor downstaging with no detrimental effect on toxicity and early clinical results have been reported among patients with a long interval between preoperative irradiation and surgery (6–8 weeks) compared with those with a short 2–week interval [22]. Since then, a 6–8 weeks “waiting” interval between preoperative radiotherapy and surgery has been preferred. We previously reported [23] a 31.6% pCR rate among patients with rectal cancer undergoing an intensified FOLFOX-based regimen with an interval of 10–12 weeks between radiotherapy completion and surgery in preoperative CCRT.

Because laparoscopic rectal surgery requires highly technically skilled surgeons experienced in minimally invasive surgery, this approach is not accepted worldwide as a standard surgical procedure for rectal cancer [30, 31]. Robotic-assisted surgery offers numerous advantages including high-definition three-dimensional vision with up to 10× magnification, articulatory instruments, a surgeon-controlled camera platform, and stable traction provided by the robotic arm. Thus, more precise dissection can be performed in the confined pelvic cavity using the robotic system. Compared with conventional laparoscopic and open surgeries for rectal cancers, the clinical and short-term oncological outcomes of robotic surgery are more favorable [32–35].

Thus far, no consensus has been reached regarding the optimal interval between the completion of radiotherapy and robotic-assisted surgery in preoperative CCRT. Thus, we conducted a retrospective study to investigate the short-term clinical and oncological outcomes of patients with stage I–III rectal cancer who underwent preoperative CCRT and robotic rectal surgery with an interval of more than 10 weeks between the completion of radiotherapy and robotic-assisted surgery. Furthermore, we compared the effects of different intervals (10–12 weeks vs ≥12 weeks) on oncological outcomes.

## Materials and methods

### Patients

**Fig 1:**
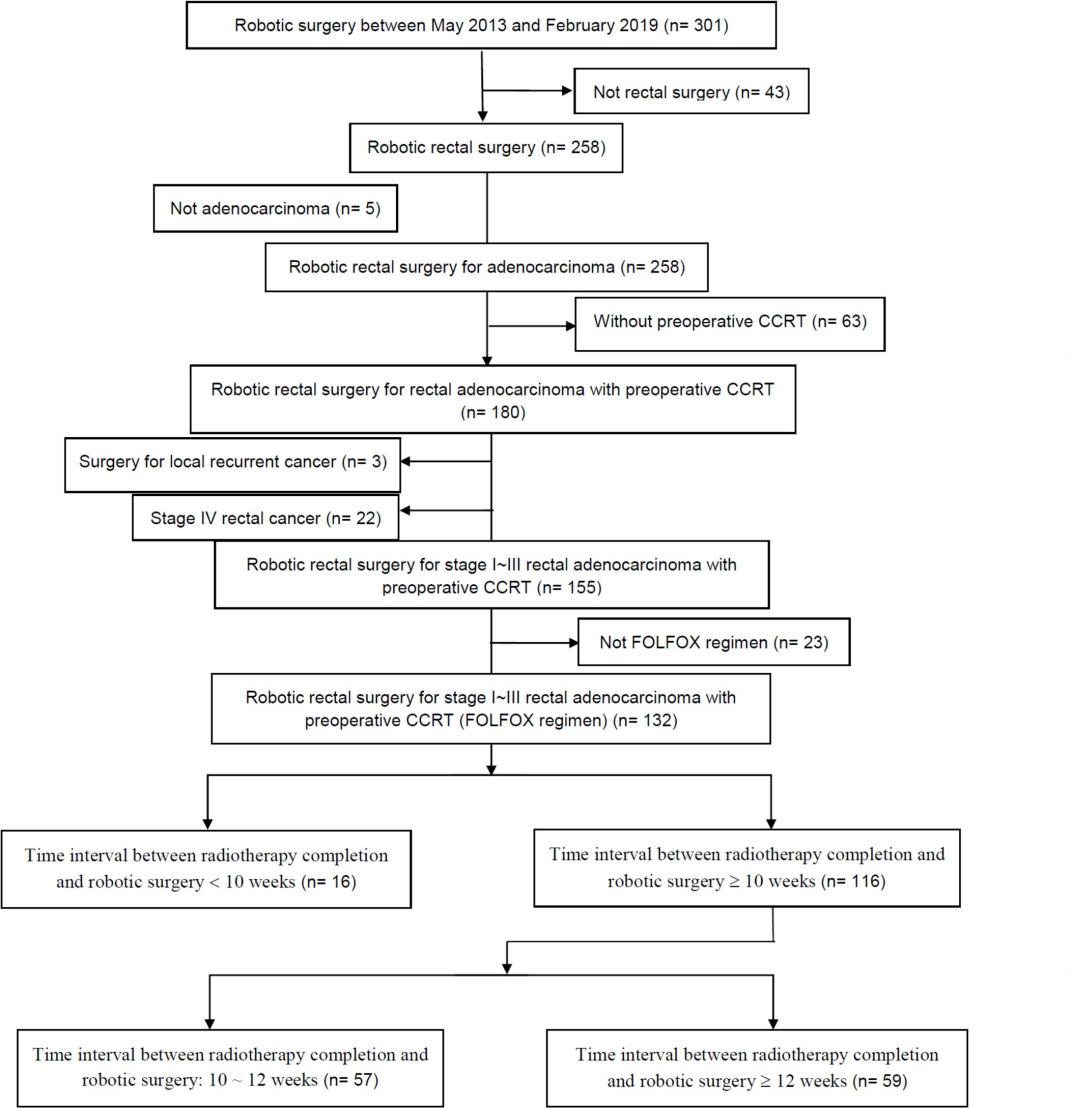
CONSORT diagram indicating the inclusion and exclusion criteria.

We retrospectively analyzed prospective data collected from a single institution, namely Kaohsiung Medical University Hospital in Taiwan. The inclusion criteria were as follows: histologically proven rectal adenocarcinoma with tumor located within 15 cm from the anal verge, clinical stage I–III, preoperative CCRT with FLOFX regimen and long-course radiotherapy (LCRT), robotic-assisted surgery, and interval between the completion of radiotherapy and robotic-assisted surgery of ≥ 10-week. In total 116 patients met the inclusion criteria and underwent preoperative CCRT followed by robotic-assisted TME with the single-docking technique using the da Vinci^®^ Si surgical system (Intuitive Surgical, Inc., Sunnyvale, CA, USA) between September 2013 and February 2019 at the abovementioned hospital (Fig 1). All data were fully anonymized before they were accessed. This study was approved by the Institutional Review Board of Kaohsiung Medical University Hospital (KMUHIRB-E(I)-20200036). All patients routinely underwent preoperative colonoscopy and abdominal and pelvic computed tomography (CT) or high definition magnetic resonance imaging (MRI) for preoperative staging. Based on the distance from the anal verge, rectal cancer was categorized into upper (11–15 cm), middle (6–10 cm), and lower (≤ 5 cm). Patients with T3, T4, or N+ rectal cancer received preoperative CCRT, including a FOLFOX (i.e., 5-fluorouracil, leucovorin, and oxaliplatin) regimen every 2 weeks and LCRT (total 5000 cGy in 25 fractions), as previously described [23]. Patients with cT2 rectal cancer within 5 cm from the anal verge also received the same preoperative CCRT.

After radiotherapy, all patients continued the biweekly FOLFOX regimen until 2–3 weeks before robotic rectal surgery. Thereafter, abdominal and pelvic CT or high definition MRI was performed for restaging. Robotic-assisted TME was performed using the single-docking technique [36] if the rectal cancer was resectable. Patients with unresectable rectal cancer underwent an additional 3–4 cycles of FOLFOX and their rectal cancer was restaged through CT scan or MRI. The clinicopathological features and perioperative parameters including age; sex; histological type; TNM (tumor, node, and metastasis) classification; vascular invasion; perineural invasion; pre-CCRT, preoperative, and postoperative serum carcinoembryonic antigen (CEA) levels; interval between the completion of preoperative radiotherapy and robotic surgery; tumor location (distance from the anal verge); American Society of Anesthesiologists (ASA) score; and body mass index (BMI) were evaluated. TNM classification was determined in accordance with the criteria of the American Joint Commission on Cancer (AJCC) and International Union Against Cancer (UICC) [37]. The tumor regression grade (TRG) was evaluated in accordance with the AJCC and College of American Pathologists regression grade [38]. Perioperative outcomes, including surgical procedures, docking time, console time, operation time, estimated blood loss, duration of the first flatus passage, duration for resuming a soft diet, duration of postoperative hospital stay, and postoperative first day visual analog scale (VAS) pain score were evaluated.

After robotic-assisted surgery, adjuvant chemotherapy was administrated, as previously reported [22]. In summary, an additional 5–6 cycles of the FOLFOX regimen were administered every 2 weeks (12 perioperative cycles in total) for patients with following risk factors: (1) ypN+ (2) positive circumferential resection margin (CRM) or distal resection margin (DRM), and (3) ypT3–4. For patients with ypT1–2N0 lesions, fluoropyrimidine-based chemotherapy was administrated for up to 6 months of perioperative chemotherapy). Patients were regularly followed-up and their clinical outcomes and survival statuses were regularly recorded as our previous studies, as previously described [36].

### Statistical analysis

All data were statistically analyzed using the Statistical Package for Social Sciences, Version 22 (SPSS Inc., Chicago, IL). All patients were followed up until their death or last follow-up. Based on the interval between the completion of radiotherapy and robotic-assisted surgery, patients were categorized into the following two groups: group A (10–12 weeks; 70–83 days) and group B (≥ 12 weeks; ≥ 84 days).The docking time was defined as the time required to position the robot and secure the robotic arms to the corresponding port sites. The console time was defined as the total duration of any robotic-assisted surgical procedure using the robotic system. The operation time was defined as the time between the initial skin incision and wound closure completion. A *P* value of <0.05 indicated statistical significance. OS was defined as the time from the date of primary treatment to the date of death from any cause or last follow-up. DFS was defined as the time from the date of primary treatment to the date of diagnosis of recurrent or metastatic disease or the date of last follow-up. OS and DFS were determined using the Kaplan–Meier method, and the log-rank test was performed to compare time-to-event distributions.

## Results

### Patient characteristics and perioperative outcomes

The baseline characteristics and perioperative outcomes of the 116 patients with rectal cancer who underwent preoperative CCRT followed by robotic-assisted surgery were summarized in Table 1. The median age and BMI of all patients were 63.5 (range, 28–88) years and 23.6 (range, 17.20–34.02) kg/m^2^, respectively. Of the 116 patients, 64 (55.2%), 34 (29.3%), 18 (15.5%) had lower, middle, and upper rectal cancers, respectively. There were 57 patients in the group A (10–12 weeks) and 59 patients in the group B (≥ 12 weeks). The median distance of the tumor from the anal verge was 5 (range, 1.0–15.0) cm, and there was no significant difference between the two groups (p = 0.215).

**Table 1:** Baseline characteristics and perioperative outcomes of 116 patients who underwent preoperative CCRT followed by robotic rectal surgery.

Characteristic		All patients (N = 116)	Time interval between radiotherapy completion and robotic surgery		
			Group A (10–12 weeks) (N = 57)	Group B (≥ 12 weeks) (N = 59)	P value
**Age (years)**					
	Mean ± SD^c^ (range)	60.8 ± 12.9 (28–88)	61.6 ± 9.8 (39–83)	60.0 ± 15.3 (28–88)	0.520
	Median	63.5	64.0	61.0	
**Gender**					0.413
	Female	43 (37.1%)	19 (33.3%)	24 (40.7%)	
	Male	73 (62.9%)	38 (66.7%)	35 (59.3%)	
**Tumor distance from anal verge (cm)**					0.215
	≦5 (Lower)	64 (55.2%)	28 (49.1%)	36 (61.0%)	
	6–10 (Middle)	34 (29.3%)	21 (36.8%)	13 (22.0%)	
	11–15 (Upper)	18 (15.5%)	8 (14.0%)	10 (17.0%)	
**Distance from anal verge (cm)**					
	Mean ± SD^a^ (range)	6.6 ± 4.4 (1.0–20.0)	6.9 ± 4.2 (1.0–15.0)	6.2 ± 4.6 (1.0–20.0)	0.403
	Median	5.0	6.0	5.0	
**Pre-CCRT**^b^ **serum CEA**^c^ **level**					0.416
	<5 ng/ml	70 (64.2%)	38 (67.9%)	32 (60.4%)	
	≥5 ng/ml	39 (35.8%)	18 (32.1%)	21 (39.6%)	
**Post-CCRT**^b^ **serum CEA**^c^ **level**					0.950
	<5 ng/ml	104 (89.7%)	51 (89.5%)	53 (89.8%)	
	≥5 ng/ml	12 (10.3%)	6 (40.5%)	6 (10.2%)	
**Post-op serum CEA**^c^ **level**					0.618^#^
	<5 ng/ml	127 (96.9%)	55 (96.5%)	57 (98.3%)	
	≥5 ng/ml	4 (3.1%)	2 (3.5%)	1 (1.7%)	
**ASA**^d^ **score**					0.729
	2	67 (57.8%)	32 (56.1%)	35 (59.3%)	
	3	49 (42.2%)		24 (40.7%)	
**BMI**^**e**^ **kg/m2**					
	Mean ± SD^a^ (range)	24.3 ± 3.5 (17.6–41.1)	24.6 ± 3.2 (19.2–33.3)	24.0 ± 3.8 (17.6–41.1)	0.331
	Median	23.6	24.2	23.3	
**Perioperative outcomes**					
**Procedure**					0.112
	LAR^f^	72 (62.1%)	40 (70.2%)	32 (54.2%)	
	ISR^g^	42 (36.2%)	17 (29.8%)	25 (42.4%)	
	APR^h^	2 (1.7%)	0 (0.0%)	2 (3.4%)	
**Protective Diverting Colostomy**					0.134
	Yes	56 (49.1%)	24 (42.1%)	32 (56.1%)	
	No	58 (50.9%)	33 (57.9%)	25 (43.9%)	
**Protective Diverting Colostomy in LAR**^f^					
	Yes	14 (19.4%)	7 (17.5%)	7 (21.9%)	0.641
	No	58 (80.6%)	33 (82.5%)	25 (78.1%)	
**Docking Time (minutes)**					
	Mean ± SD^a^ (range)	4.0 ± 1.4 (3.0–11.0)	4.1 ± 1.2 (3.0–8.0)	4.5 ± 1.6 (3.0–11.0)	0.125
	Median	4.0	4.0	4.0	
**Console Time (minutes)**					
	Mean ± SD^a^ (range)	186.6 ± 47.8 (110.0–365.0)	181.0 ± 43.8 (120.0–340.0)	191.9 ± 51.3 (110.0–365.0)	0.226
	Median	175.0	175.0	180..	
**Operation Time (minutes)**					
	Mean ± SD^a^ (range)	303.4 ± 70.1 (200.0–620.0)	295.6 ± 56.3 (200.0–465.0)	314.8 ± 80.7 (200.0–620.0)	0.146
	Median	295.0	290.0	300.0	
**Estimated blood loss (mL)**		±			
	Mean ± SD^a^ (range)	1109.7 ± 135.6 (15.0–11050.0)	189.4 ± 82.8 (20.0–550.0)	129.2 ± 170.5 (15.0–1050.0)	0.117
	Median	770.0	50.0	77.5	
**Duration of first flatus passage (day)**					
	Mean ± SD^a^ (range)	1.7± 1.0 (1.0–10.0)	1.6± 0.7 (1.0–3.0)	1.7 ± 1.3 (1.0–10.0)	0.552
	Median	2.0	1.5	2.0	
**Duration for resuming a soft diet (day)**					
	Mean ± SD^a^ (range)	3.8 ± 1.3 (2.0–12.0)	3.7 ± 1.0 (2.0–8.0)	3.9 ± 1.6 (2.0–12.0)	0.389
	Median	3.0	3.0	4.0	
**Post operative hospital stay (day)**					
	Mean ± SD^a^ (range)	7.1 ± 4.3 (4.0–46.0)	6.6 ± 2.2 (4.0–18.0)	7.6 ± 5.6 (5.0–46.0)	0.236
	Median	6.0	6.0	6.0	
**Post-operative first day pain score**					
	Mean ± SD^a^ (range)	3.3 ± 1.54 (0.0–7.0)	3.1 ± 1.4 (0.0–7.0)	3.4 ± 1.4 (1.0–7.0)	0.286
	Median	3.0	3.0	3.0	

^**a**^ SD standard deviation

^**b**^ CCRT concurrent chemoradiotherapy

^**c**^ CEA carcinoembryonic antigen

^**d**^ ASA American Society of Anesthesiologists

^**e**^ BMI body mass index

^**f**^ LAR low anterior resection

^**g**^ ISR: intersphenteric resection

^**h**^APR abdominoperineal resection.

* P value < 0.05

^#^ Fisher exact test.

The most frequent surgical procedure was low anterior resection (LAR) (72/116, 62.1%), which was performed in 40 (70.2%) of group A patients and 32 (54.2%) of group B patients. Intersphenteric resection (ISR) with coloanal anastomosis was performed in 32 (33.7%) patients, which was performed in 17 (29.8%) of group A patients and 25 (42.4%) of group B patients. Abdominoperineal resection (APR) was performed in 2 (3.4%) of group B patients. Protective diverting loop transverse colostomy was performed for 56 (49.1%) patients, including 42 and 14 patients who underwent ISR and LAR, respectively. Furthermore, the sphincter preservation rate of all patients was determined from among 114 of 116 patients (98.3%). No significant differences between the two groups were observed in console time, operation time, estimated blood loss, duration of postoperative first flatus passage, duration for postoperative resuming a soft diet, postoperative hospital stay, and postoperative first day pain score (all *P*>0.05).

### Pathological and oncological outcomes

**Fig 2:**
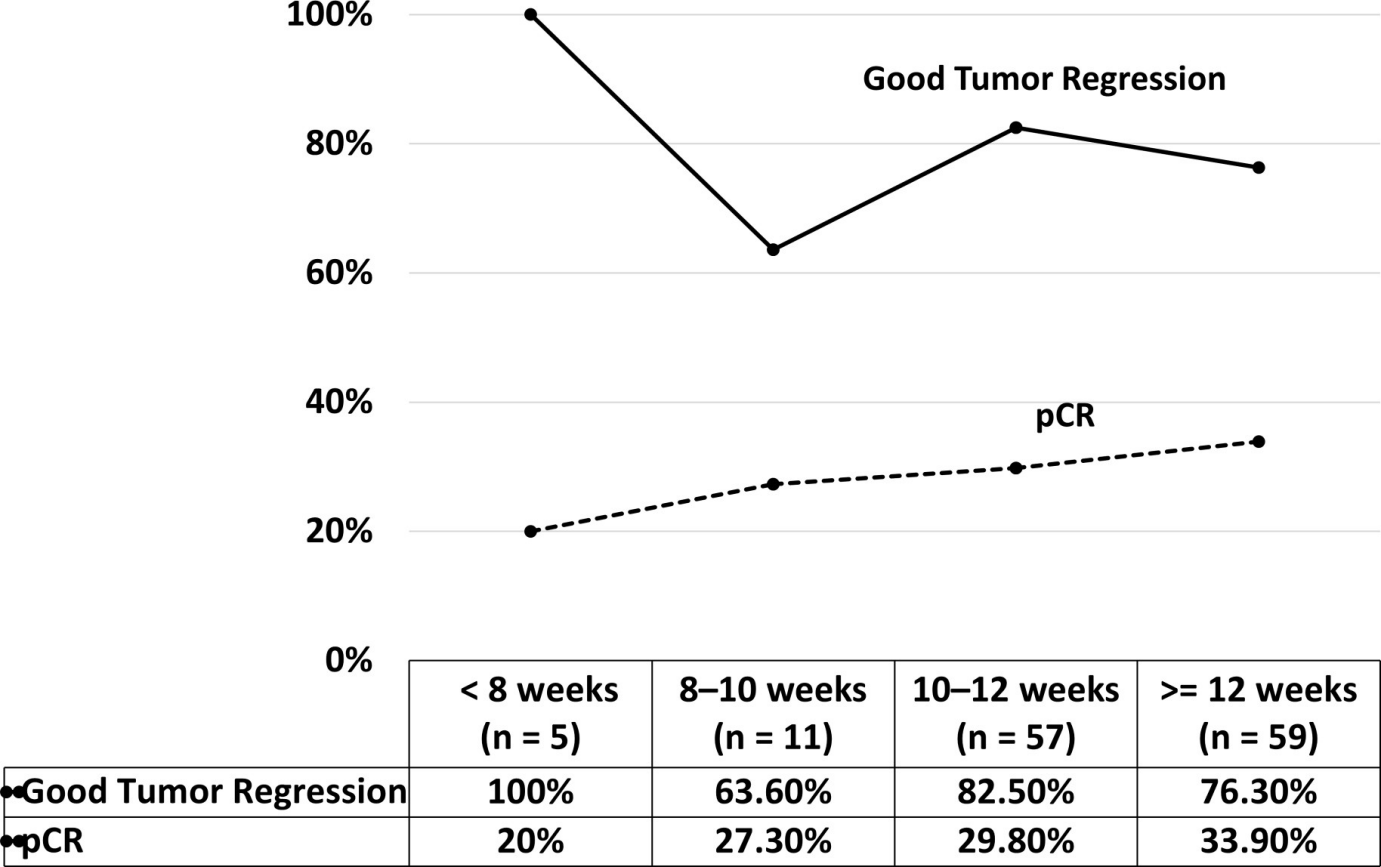
Pathologic complete response rate and good tumor regression rate at different intervals.

The pathological characteristics and oncological outcomes of all 116 patients are listed in Table 2. Preoperative clinical staging demonstrated that the majority of the patients with LARC were T3 in 91 (76.5%) patients, T4 in 19 (15.2%) patients, or N+ in 63 (77.6%) patients. T4 lesions were more common in group B than group A (23.7% vs 8.7%, respectively), but the difference was not significant (p = 0.092). No significant differences were observed in clinical T stage, N stage, and AJCC stage (all *P*>0.05). Furthermore, pCR (ypT0) was noted in 37 (31.9%) patients. Good tumor regression (TRG 0 and 1) was noted in 92 (79.3%) patients, and it was more common in group A than group B (82.5% vs 76.3%, respectively), but the difference was not significant (*P* = 0.411). Combined analysis of the pCR rate and the good tumor regression rate among all 132 patients with clinical stage I–III disease undergoing preoperative CCRT with FOLFOX regimen and robotic-assisted surgery revealed an optimal interval of 10–12 weeks between the completion of radiotherapy and robotic-assisted surgery, because no clear benefits were observed for interval ≥12 weeks (Fig 2). The pCR rate and good tumor regression rate at the time interval of 10–12 weeks were 29.8% and 82.5%, respectively. DRM and CRM were positive in 1 (0.9%) and 2 (1.7%) patients, respectively. Therefore, R0 resection rate for primary rectal cancer was 97.4% (113/116 patients).

**Table 2:** Pathologic characteristics and oncological outcomes of 116 patients who underwent preoperative concurrent chemoradiotherapy followed by robotic rectal surgery.

Characteristic		All patients (N = 116)	Time interval between radiotherapy completion and robotic surgery		
			Group A (10–12 weeks) (N = 57)	Group B (≥ 12 weeks) (N = 59)	P value
**Preoperative clinical staging**					
**Tumor depth**					0.092
	T2	6 (5.2%)	3 (5.3%)	3 (5.1%)	
	T3	91 (76.5%)	49 (86.0%)	42 (71.2%)	
	T4	19 (15.2%)	5 (8.7%)	14 (23.7%)	
**Lymph Node metastasis**					0.540
	N0	26 (22.4%)	14 (24.6%)	12 (20.3%)	
	N1	63 (54.3%)	28 (49.1%)	35 (59.4%)	
	N2	27 (23.3%)	15 (26.3%)	12 (20.3%)	
**AJCC**^a^ **Stage (Clinical)**					0.771
	I	3 (2.6%)	2 (3.5%)	1 (1.7%)	
	II	23 (19.8%)	12 (21.1%)	11 (18.6%)	
	III	90 (77.6%)	43 (75.4%)	47 (79.7%)	
**Postoperative pathological outcomes**					
**Tumor size**					1.000^#^
	< 5 cm	111 (95.7%)	55 (96.5%)	56 (94.9%)	
	≥ 5 cm	5 (4.3%)	2 (3.5%)	3 (5.1%)	
**Tumor size (cm)**					
	Mean ± SD^c^ (range)	1.8 ± 1.8 (0.0–8.0)	2.3 ± 1.8 (0.0–5.8)	1.8 ± 1.7 (0.0–8.0)	0.273
	Median	1.5	1.5	1.5	
**Tumor depth**					0.655
	**T0**	37 (31.9%)	17 (29.8%)	20 (33.9%)	
	**Tis**	1 (0.9%)	1 (1.8%)	0 (0.0%)	
	**T1**	10 (8.6%)	5 (8.8%)	5 (8.5%)	
	**T2**	29 (25.0%)	14 (24.6%)	15 (25.4%)	
	**T3**	37 (31.9%)	20 (35.0%)	17 (28.8%)	
	**T4**	2 (1.7%)	0 (0.0%)	2 (3.4%)	
**Lymph Node metastasis**					0.193
	N0	95 (81.9%)	43 (75.4%)	52 (88.1%)	
	N1	17 (14.7%)	11 (19.3%)	6 (10.2%)	
	N2	4 (3.4%)	3 (5.3%)	1 (1.7%)	
**AJCC**^a^ **Stage (Pathologic)**					0.307
	0	31 (1.7%)	18 (31.6%)	19 (32.2%)	
	I	32 (27.6%)	13 (22.7%)	19 (32.2%)	
	II	26 (22.5%)	12 (21.1%)	14 (23.7%)	
	III	21 (18.0%)	14 (24.6%)	7 (11.9%)	
**Down Stage of T Stage**					0.485
	Down Stage	84 (72.4%)	40 (70.2%)	44 (74.6%)	
	Unchanged	31 (26.7%)	17 (57.8%)	14 (23.7%)	
	Up Stage	1 (0.9%)	0 (0.0%)	1 (1.7%)	
**Down Stage of N Stage**					0.476
	Down Stage	82 (71.3%)	38 (67.9%)	44 (74.6%)	
	Unchanged	32 (27.8%)	17 (30.4%)	15 (25.4%)	
	Up Stage	1 (0.9%)	1 (1.7%)	0 (0.0%)	
**Down Stage of AJCC**^a^ **Stage**					0.073
	Down Stage	100 (86.2%)	45 (78.9%)	55 (93.2%)	
	Unchanged	15 (12.9%)	11 (19.3%)	4 (6.8%)	
	Up Stage	1 (0.9%)	1 (1.8%)	0 (0.0%)	
**Tumor Regression Grade**					0.713
	0	37 (32.8%)	18 (31.6%)	20 (33.9%)	
	1	55 (46.6%)	29 (50.9%)	25 (42.4%)	
	2	16 (13.8%)	6 (10.5%)	10 (16.9%)v	
	3	8 (6.9%)	4 (7.0%)	4 (6.8%)	
**Tumor Regression**					0.411
	Good (0+1)	92 (79.3%)	47 (82.5%)	45 (76.3%)	
	Poor (2+3)	24 (20.7%)	10 (17.5%)	14 (2.7%)	
**Harvested Lymph Node**					
	Mean ± SD^c^ (range)	9.8 ± 5.21 (0–30)	9.3 ± 5.1 (2–23)	9.9 ± 5.2 (0–30)	0.682
	Median	9.0	10.0		
**Positive Lymph Node**					
	Mean ± SD^c^ (range)	0.5 ± 2.3 (0–24)	0.3 ± 0.6 (0.0–2.0)	0.5 ± 2.4 (0.0–24.0)	0.725
	Median	0.0	0.0	0.0	
**Harvested Apical Node**					
	Mean ± SD^c^ (range)	2.2 ± 1.9 (0–10)	1.4 ± 1.4 (0.0–3.0)	2.4 ± 2.0 (0.0–10.0)	0.051
	Median	2.0	2.0	2.0	
**Positive Apical Node**					
	Mean ± SD^c^ (range)	0.02 ± 0.12 (0–1)	± 0.0 (0.0–0.0)	0.02 ± 0.13 (0.0–1.0)	0.600
	Median	0.0	0.0	0.0	
**Vascular invasion**					0.198^#^
	No	104 (95.4%)	49 (92.5%)	55 (98.2%)	
	Yes	5 (4.6%)	4 (7.5%)	1 (1.8%)	
**Perineural invasion**					0.728
	No	91 (84.3%)	44 (83.0%)	47 (85.5%)	
	Yes	17 (15.7%)	9 (17.0%)	8 (14.5%)	
**Distance of proximal resection margin (cm)**					0.817
	Mean ± SD^c^ (range)	6.0 ± 2.6 (1.0–19.0)	6.1 ± 2.4 (2.0–11.0)	6.4 ± 5.6 (2.5–12.5)	
	Median	6.0	5.0	6.0	
**Distance of distal resection margin** (cm)					
	Mean ± SD^c^ (range)	2.4 ± 1.6 (0.1–8.1)	2.2 ± 1.2 (0.1–5.0)	2.4 ± 1.7 (1.0–8.0)	0.653
	Median	2.2	2.5	2.0	
**Circumferential resection margin**					
	Free	114 (98.3%)	57 (100.0%)	57 (96.6%)	0.496^#^
	Positive	2 (1.7%)	0 (0.0%)	2 (3.4%)	
**Distal resection margin**					
	Free	115 (99.1%)	57 (100.0%)	58 (98.3%)	1.000^#^
	Positive	1 (0.9%)	0 (0.0%)	1 (1.7%)	
**Resection Degree of Primary tumor**					
	R0	113 (97.4%)	57 (100.0%)	56 (94.9%)	0.244^#^
	R1	3 (2.6%)	0 (0.0%)	3 (5.1%)	
**Oncological outcomes**					
**Follow-up periods** (months, median) (range)		30.0 (11.4–83.4)	28.3 (11.4–67.5)	30.4 (12.7–83.4)	
**Post-op relapse**					0.093
	No	90 (77.6%)	48 (84.2%)	42 (71.2%)	
	Yes	26 (22.4%)	9 (15.8%)	17 (28.8%)	
**Post-op locoregional Relapse**					0.119^#^
	No	112 (96.6%)	57 (100.0%)	55 (93.2%)	
	Yes	4 (3.4%)	0 (0.0%)	4 (6.8%)	
**Post-op distant Relapse**					0.391
	No	94 (81.0%)	48 (84.2%)	46 (78.0%)	
	Yes	22 (19.0%)	9 (15.8%)	13 (22.0%)	
**Post-op mortality**					0.125^#^
	No	104 (89.7%)	54 (94.7%)	50 (84.7%)	
	Yes	12 (10.3%)	3 (5.3%)	9 (15.3%)	
**DFS**^d^ (Median, Range) (Months)		27.6 (8.1–83.4)	27.6 (11.4–67.5)	27.6 (8.1–83.4)	
**OS**^e^ (Median, Range) (Months)		30.0 (11.4–83.4)	28.3 (11.4–67.5)	30.4 (12.7–83.4)	

^**a**^AJCC American Joint Commission on Cancer

^**b**^ NA not available

^**c**^ SD standard deviation

^**d**^ DFS disease-free survival

^**e**^ OS overall survival

^#^ Fisher exact test.

**Fig 3:**
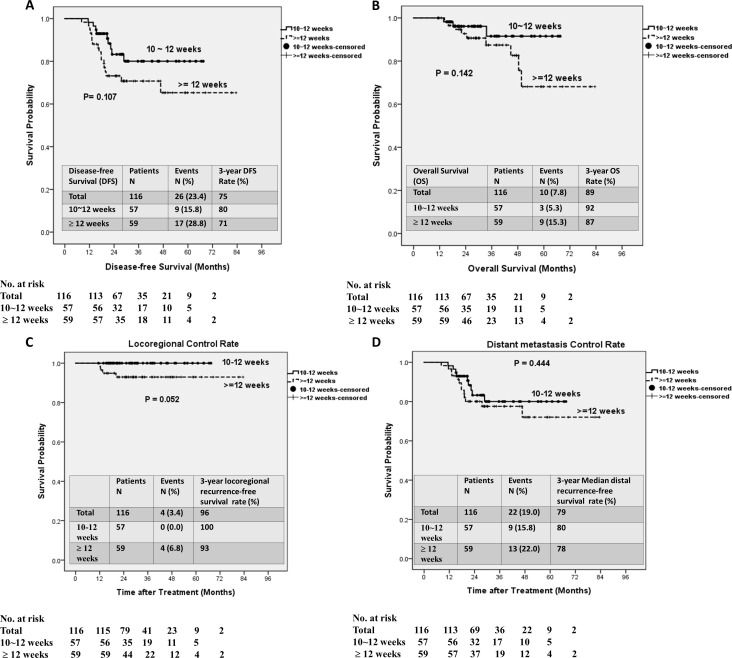
Kaplan–Meier survival curves for different intervals the completion of between radiotherapy and robotic-assisted surgery. (A) Disease-free survival. (B) Overall survival. (C) Locoregional control rate. (D) Distant metastasis control rate.

The median follow-up duration of 116 patients after primary treatment was 30.0 months (range: 11.4–83.4 months). Twenty-six (22.4%) patients experienced postoperative relapse, including local recurrence in 4 (3.4%) and distant metastases in 22 (19.0%) patients. Postoperative relapse was slightly more common in group B than in group A, but the difference was not significant (28.8% vs 15.8%, *P* = 0.093). Furthermore, 12 (10.3%) patients died after surgery; post-surgery death was slightly more common in group B than in group A, but this difference was not significant (15.3% vs 5.3%, *P* = 0.125). At a median follow-up duration of 30.0 months, 3-year DFS rate was 75% (Fig 3A) and the 3-year OS rate was 89% (Fig 3B). Moreover, the 3-year locoregional control rate was 96% (Fig 3C), and the 3-year distant metastasis control rate was 79% (Fig 3D). The 3-year DFS rate was higher in group A than in group B (80% vs 71%, Fig 3A), the 3-year OS rate was higher in group A than group B (92% vs 87%, Fig 3B), the 3-year locoregional control rate was higher in group A than group B (100% vs 93%, Fig 3C), and the 3-year distant metastasis control rate was higher in group A than in group B (80% vs 78%, Fig 3D); however, none of these differences were significant (all *P* > 0.05).

### Postoperative complications

Postoperative complications are summarized in Table 3. Postoperative complications were observed in 27 patients (23.3%), and no significant difference between the two groups were observed (*P* = 0.578). Anastomosis leakage was observed in 4 (3.4%) patients who underwent LAR with the double-stapled anastomosis, and loop transverse colostomy was subsequently performed. Two patients (1.7%) experienced anastomosis bleeding and recovered after conservative treatment. Eight (7.0%) patients experienced stenosis of coloanal anastomosis and underwent dilation using a colonoscope. Intraabdominal abscess were observed in three patients, and CT-guided pigtail drainage was subsequently performed in two patients. Based on the Clavien-Dindo Classification system, all postoperative ileus, urinary retention, central venous catheter infection, neck cellulitis, and pulmonary complications were of grade I, and the patients displayed an uneventful recovery course after conservative treatment. Moreover, no 30-day hospital mortality occurred.

**Table 3:** Postoperative complications in 116 patients who underwent preoperative concurrent chemoradiotherapy followed by robotic-assisted surgery.

Complications	All patients (N = 116)	Time interval between radiotherapy completion and robotic surgery		
		Group A (10–12 weeks) (N = 57)	Group B (≥ 12 weeks) (N = 59)	P value
Total complications				0.578
No	89 (76.7%)	45 (78.9%)	44 (74.6%)	
Yes	27 (23.3%)	12 (21.1%)	15 (25.4%)	
Type of complications				
Anastomosis leakage	4 (3.4%)	2	2	
Anastomosis bleeding	2 (1.7%)	2	0	
Anastomosis Stenosis	8 (7.0%)	2	6	
Ileus	5 (4.3%)	2	3	
Pulmonary complication	2 (1.7%)	0	2	
Sexual dysfuction	1 (0.9%)	1	0	
Urinary retention	3 (2.6%)	1	2	
Central venous catheter infection	1 (0.9%)	1	1	
Neck cellulitis	1 (0.9%)	1	1	

## Discussion

This study revealed the short-term clinical and oncological outcomes of 116 patients with stage I–III rectal cancer who underwent preoperative CCRT and robotic rectal surgery with a ≥10 weeks interval between the completion of radiotherapy and robotic-assisted surgery. Because this was a retrospective study, we did not calculate the sample size or power to reveal the real-world evidence of our clinical practices. These patients had a relatively high pCR rate (31.9%), R0 resection rate (97.4%), and sphincter preservation rate (98.3%). However, their overall postoperative complication rate (23.3%) and 3-year short-term oncological outcomes were consistent with those previously reported [39–43]. No significant differences were observed between the two groups in short-term oncological outcomes; hence, the time interval of 10–12 weeks between the completion of radiotherapy and robotic-assisted surgery can be considered as a safe interval, because no clear benefits were observed beyond this interval.

Chemotherapy with a FOLFOX regimen was administrated during preoperative CCRT and was continued upon the completion of radiotherapy. In the German CAO/ARO/AIO-04 study, Rödel *et al*. evaluated the effect of the addition of oxaliplatin upon preoperative CRT followed by TME surgery 5–6 weeks after completion of CRT, and they reported a R0 resection and pCR rates of 94% and 17%, respectively when combining fluorouracil with oxaliplatin, and 95% and 13%, respectively, with fluorouracil alone [13, 14]. They concluded that combinatorial treatment with oxaliplatin and a fluorouracil-based treatment was well-tolerated and increased the pCR rate. In a multicenter, phase II randomized controlled trial [18, 29], Garcia-Aguilar *et al*. evaluated the effect of the supplementation of mFOLFOX6 cycles between chemoradiation and surgery. The pCR rate increased from 18% to 38% upon supplementation of 2, 4, and 6 mFOLFOX6 cycles; the risk of postoperative complications and surgical difficulty remained unaltered. Conversely, in a phase III randomized trial, Gérard *et al*. reported non-significant increases in the pCR rate from 13.9% to 19.2% and in CRM negative resection rate, from 87.3% to 92.3%, upon supplementation of oxalipatin [44], indicating that oxaliplatin supplementation did not have clinical benefits. In a systemic review and meta-analysis study, Yang *et al*. reported that administration of OX/FU regimen resulted in a significant higher pCR rate compared with the FU regimen [39]; however, no significant differences were observed between the two treatment regimens in the R0 resection rate and the positive CRM rate. Another meta-analysis of eight randomized control trials also reported a higher pCR rate in the oxaliplatin-based regimen group [40].

Preoperative rather than postoperative radiotherapy has been reported to reduce the LR rate and complication rate [4–8]. Preoperative radiotherapy may induce tissue swelling and local inflammation, which requires time to subside. Therefore, a longer interval between radiotherapy completion and surgery for higher degrees of tumor shrinkage. This study reported good tumor regression rate of 79.3% and pCR of 31.9% of rate with an interval ≥10 weeks between the completion of radiotherapy and robotic-assisted surgery. Furthermore, this study revealed a high R0 resection rate (97.4%). Garcia-Aguilar *et al*. reported that a modest increase in the pCR rate without an increase in postoperative complications may result from intensive neoadjuvant therapy and delayed surgery (≥10 weeks vs 6–8 weeks) [18, 29]. In a Chinese FOWARC randomized controlled trial, Deng *et al*. reported a pCR rate of 27.5%, good tumor regression rate of 68.5%, and R0 resection rate of 89.9% upon administration of an mFOLFOX6 regimen during preoperative CCRT with a 52-day median interval between the completion of chemoradiotherapy and TME [15]. Sloothaak *et al*. [41] analyzed the database of the Dutch Surgical Colorectal Audit to evaluate the optimal interval between neoadjuvant CRT and surgery, and they reported the highest pCR rate (18.0%), T-downstaging rate (55.2%), and N-downstaging rate (55.2%) among patients with an interval of 10–11 weeks from the completion of CCRT. Lichthardt *et al*. retrospectively analyzed the German StuDoQ|Rectalcarcinoma registry and reported a prolonged interval (≥8 and 8–10 weeks vs <6 and 6–8 weeks) between the completion of CCRT and oncological resection in patients with LARC is potentially beneficial in increasing the pCR and TRG rates without increasing perioperative morbidity [42]. Moreover, because of the relatively high T-downstaging rate, good tumor regression rate, and pCR rate observed in the current study, the positive CRM and DRM rates were 1.7% and 0.9%, respectively. However, the positive CRM rate reported by Lim *et al*. was 2.8% [28]; that reported in the GRECCAR-6 study was 10.8% [26, 27]; and that reported by Law *et al* was 3.9%–6.6% [45]. Moreover, the overall positive DRM rate was 2.1% in the GRECCAR-6 study [26, 27].

This study revealed that intensified multimodality preoperative treatment (extended FOLFOX chemotherapy, LCRT, and a longer interval between the completion of radiotherapy and surgery) resulted in a pCR (31.9%) higher than those observed in previous studies (8%–38%; <20% in most studies) [11–18]. Although 98 (84.5%) patients had middle-to-low rectal cancers in the current study, a comparable sphincter preservation rate (98.3%), a favorable overall CRM positive rate (1.7%), a relatively low protective diverting ostomy creation rate (49.1%), and a comparable anastomosis leakage rate (3.4%) were noted [45–49]. Furthermore, the perioperative outcomes and overall postoperative complication rate (23.3%) were also comparable to previously reported values [45–49]. Therefore, in the present study, our intensified multimodality preoperative treatment did not increase the difficulties associated with robotic-assisted rectal surgery and postoperative complication rates. Furthermore, with a the median follow-up duration among the 116 patients from the primary treatment of 30.0 months (range: 11.4–83.4 months), this study reported a 75.0% 3-year DFS, 89% 3-year OFS, 96% 3-year locoregional control rate, and 79% 3-year distant metastasis control rate. These oncological outcomes are comparable with those of previous studies [45–48]. In the present study, the time interval between radiotherapy completion and surgery was not considered more a “waiting time”, but rather an “extending treatment time”.

Because pCR and good tumor regression are indicators of favorable oncological outcomes for preoperative CCRT [12, 38, 50], we analyzed the effects of time intervals on these factors. The pCR rate was the highest among patients with an interval of ≥ 12 weeks (33.9%), whereas good tumor regression was superior among patients with an interval of 10–12 weeks (82.5%). Although good tumor regression was optimal among patients with an interval of < 8 weeks (100%), the number of patients in this group was too small (n = 8). Furthermore, preferable oncological outcomes including DFS, OS, 3-year locoregional control, and 3-year distant metastasis control were observed among patients with an interval of 10–12 weeks compared with those with an interval of ≥ 12 weeks. These differences were nonsignificant, which might have resulted from small sample size. Therefore, based on the present results, 10–12 weeks may be considered as a safe interval between the completion of radiotherapy and robotic-assisted rectal surgery.

This study had several limitations. First, this was a single-center retrospective study with a small sample of 116 patients which probably was probably insufficient for highlighting differences between the two groups. Second, we did not evaluate the toxicity rates of preoperative CCRT; however, no treatment-related death occurred in and all patients completed the preoperative CCRT and postoperative chemotherapy. Third, the follow-up interval was relatively short, with a 30-month median follow-up duration; thus, only short-term (3-year) survival and oncological outcomes were documented. Fourth, we did not evaluate the postoperative outcomes of urinary, sexual functions, or anal functions.

## Conclusions

An intensified multimodal preoperative treatment with a longer interval (≥10 weeks) between the completion of radiotherapy and robotic-assisted surgery potentially increases the pCR rate and the good tumor regression rate; furthermore, preferable oncological outcomes were observed without any effects on the overall complication rate among patients with an interval of 10–12 weeks. Therefore, robotic-assisted surgery after a 10–12-week is safe and feasible for patients with rectal cancer undergoing preoperative CCRT. However, further studies with a longer follow-up duration are required to investigate the long-term oncological outcomes. Moreover, further prospective randomized clinical trials are required to validate the present results.

## Supporting information

S1 Data (XLSX)

S2 Data (XLSX)
